# Intestinal and hepatic coccidiosis among rabbits in Yogyakarta, Indonesia

**DOI:** 10.14202/vetworld.2019.1256-1260

**Published:** 2019-08-15

**Authors:** Penny Humaidah Hamid, Sigit Prastowo, Yuli Purwandari Kristianingrum

**Affiliations:** 1 Faculty of Veterinary Medicine, Universitas Gadjah Mada, Yogyakarta, Indonesia; 2 Department of Animal Science, Universitas Sebelas Maret, Indonesia

**Keywords:** coccidiosis, Indonesia, rabbit

## Abstract

**Background and Aim:**

The attention to rabbit meat production in Indonesia is comparatively less to other farm animals such as cattle and poultry industries. However, future prospect of rabbit to be seriously industrialized seemed quite promising due to rabbit is highly productive and has short reproduction cycle as well as generation interval. One of the diseases infecting many rabbits is coccidiosis caused by protozoan parasite, *Eimeria* spp. The infectious stage of *Eimeria* spp. presents ubiquitously in the environment and increases the risk of parasite transmission. Preventive methods such as vaccination are not yet fully developed, while sporadic treatment is not efficiently reduce the cases. In this study, *Eimeria* spp. infecting rabbits in Yogyakarta Province, Indonesia, were investigated with the aim for precise diagnosis to determine targeted treatment and as a baseline epidemiological data from rabbit in Indonesia.

**Materials and Methods:**

Sample collection was performed randomly for 3 months, from March 2017 to May 2017 and covered areas in Yogyakarta, Indonesia. A total of 750 samples were collected. *Eimeria* species identification was determined morphologically from the samples after sporulation in 2.5% potassium dichromate byCOCCIMORPH.

**Results:**

Ten species of *Eimeria* spp. were identified in this study from the positive samples (527/750; 70.3%). *Eimeria flavescens* was present in 80% of the positive samples, *Eimeria coeciola* in 78%, *Eimeria perforans* in 61%, *Eimeria exigua* in 37%, *Eimeria media* in 33%, *Eimeria stiedae* in 31%, *Eimeria irresidua* in 12%, *Eimeria magna* in 11%, *Eimeria intestinalis* in 10%, and *Eimeria piriformis* in 10%. Coinfection as noted in 80% of the positive samples with 2-6 species in a specimen. *E. flavescens* and *E. coeciola* were the most prevalent among all *Eimeria* spp. (p≤0.0001).

**Conclusion:**

*Eimeria* spp. is detected in high prevalence among rabbit in Yogyakarta, Indonesia, with commonly occurs in mixed infections. In this paper, we describe *Eimeria* spp. that are circulating in Indonesia and present it as updated information to farmers and veterinarians. To the best of our knowledge, we provide the first information about rabbit coccidiosis in Indonesia.

## Introduction

The rabbit is a small animal belonging to lagomorphs, according to the digestive system, it is categorized as pseudoruminant that able to utilize large amounts of roughages by having large cecum and large intestine for microbial digestion. Further, rabbit could be easily raised for high-quality meat which known to be healthier than meat from other ruminant animals since its content is high in polyunsaturated fatty acids and many essential amino acids. Rabbit meat also provides high calories, but low of low-density lipoproteins and cholesterol levels, which is attracting more consumers with healthy lifestyle trends [1]. The world global production of rabbit meat is increasing with a rise in production from 1,224,186 tons in 2010 to 1,428,085 tons in 2016 [2]. The largest production of rabbit meat in Asia is derived from China, which supplies 80% of Asia’s demands [2]. In Europe, around 180 million rabbits are reared to supply meat consumption demands. Rabbits rank sixth for supporting meat consumption after poultry, laying hens, trout, salmon, and pigs [3].

At present, the attention to rabbit meat production in Indonesia is less when compared with other farm animals such as cattle and poultry. The total rabbit population in 2018 was 1,251,018 and was mostly concentrated on Java Island [4]. Indonesia may also consider heightening awareness of using rabbit as a source of good meat by reflecting on both the rabbit population and knowledge of the commercial industry in other countries. Since meat production is always below its large national demands, Indonesia has had to import cattle meat from aboard for years [5]. The managerial problem of the cattle industry leads to losses of meat productivity and, therefore, increases meat prices in society. Self-sufficient production of meat from cattle is not possible, mainly due to animal health issues and some managerial problems, coupled with losses in productivity [6]. Rabbit as a meat source can serve as a potential alternative to support the meat demand of 250 million people besides the meat from cattle, chicken, and fishes. Rabbit husbandry seems quite promising because rabbit is highly productive in terms of the number of resulted offspring, short gestation and lactation periods, and great prolificacy. It may produce 30-40 young weaned offspring per doe annually in tropical climate with semi-intensive reproduction management [7]. Moreover, the investment and labor costs to initiate rabbit husbandry are relatively small and can be handled by most family members in the backyard. In addition to that, rabbits are easy to transport and market for food, fur, skin, and do not need a large amount of feed and housing space.

Coccidiosis is persistently one of the most important primary causes of digestive disease in fattening rabbits [8]. Coccidiosis not only has a direct impact on performance but also acts in synergy with epizootic rabbit enteropathy [8]. Rabbit coccidiosis or eimeriosis is caused by the apicomplexan parasite, *Eimeria* spp. To date, 17 different *Eimeria* species have been described that infect rabbits worldwide [9]. *Eimeria* spp. always present on rabbit farms, are found ubiquitously in the environment, and are virtually impossible to eradicate. Therefore, knowledge of coccidiosis remains of utmost importance. *Eimeria* spp. infects the host orally with the infective stage, i.e., a sporulated oocyst. The sporozoite enters the endothelial or epithelial cell of the host intestine and forms schizogony processes [10]. The newly developed merozoites will infect neighboring cells and undergo gametogony. Later, oocysts will be passed with fecal samples and develop further sporogony process in the environment [10]. During this schizogony process and cell rupture due to merozoites egress, clinical manifestations can be observed due to disorders in feed metabolism. *Eimeria stiedae* infections are characterized by endogenous development within the rabbit epithelium of the bile duct. This specific site of development stage results in specific pathological changes as known as hepatic coccidiosis.

A trial of vaccination using selected precocious lines of *Eimeria* spp. has been introduced to prevent rabbit coccidiosis with promising results on a laboratory scale [11,12]. However, production on a large or commercial scale to be applied in the field is still far away due to the time required by processes including optimization, registration, safety, and distribution to customers. It is not recommended to perform sporadic treatment since it does not efficiently reduce environmental contamination with infectious oocysts and potent parasite transmission. In Indonesia, many farmers are not familiar with infectious gastrointestinal diseases mainly caused by protozoa in rabbits. Coccidiosis usually occurs without any clinical or nonspecific symptoms. The correct diagnosis is the critical point in choosing a treatment and reducing coccidiosis cases in the field.

In this study, *Eimeria* spp. infecting rabbits in Yogyakarta Province, Indonesia, were investigated with the aim for precise diagnosis to determine targeted treatment and as a baseline epidemiological data from rabbit in Indonesia.

## Materials and Methods

### Ethical approval

Ethical Clearance regarding necropsies of hepatic coccidiosis was issued by “Laboratorium Penelitian dan Pengujian Terpadu, Universitas Gadjah Mada”, Indonesia ([LPPT UGM], no. 00047/04/LPPT/IV/2017.

### Sample collection

Sample collection was performed randomly for 3 months, from March to May 2017 in several districts of Kulonprogo, Bantul, and Sleman, Yogyakarta, Indonesia (included in the Special Region of Yogyakarta, 7°43’57.0”S and 110°20’11.0”E). A total of 750 fecal samples were collected in plastic containers and stored at 4°C until the time of examination. In this study, feces were collected from rectums of rabbits reared in a group and fecal samples from individually caged rabbits. Along with that, rabbits with fecal samples containing *E. stiedae* oocysts were identified, and 23 rabbits were necropsied to confirm with histopathology analysis.

### Fecal sample processing

Fecal samples were sedimented by short centrifugation (36× g, 5 min). After sedimentation, the water was discharged and saturated NaCl was added to float the oocysts. Parasitological objects were observed microscopically under 400×. Oocysts per gram (OPG) of feces were counted for samples using the McMaster technique [13]. Briefly, 2 g of each sample was mixed in 30 ml of salt solution at room temperature. Large debris was removed by pouring the fecal sample through a wire mesh. Then, 0.5 ml of the suspension was added to a McMaster slide. Both chambers were observed under a light microscope using 100×. Oocysts were counted by multiplying the total number of oocysts by 50. Since the sample weights were varied and sampling was performed once per individual rabbit, we did OPG counts on samples with sufficient amounts only (257 from total of 527 positive samples). Identification of *Eimeria* species was performed after sporulation of positive samples in 2.5% potassium dichromate at room temperature. *Eimeria* species were identified under a microscope at 400×. *Eimeria* species identification was determined morphologically from sporulated oocysts by COCCIMORPH (http://www.coccidia.icb.usp.br/coccimorph) software [14].

### Determination of hepatic samples of infected rabbits

The livers from *E. stiedae*-infected rabbits were processed using hematoxylin-eosin staining to evaluate hepatic sample lesions.

### Statistical analysis

In this study, we observed several parameters, namely, the prevalence and species variation of coccidiosis due to *Eimeria* spp. in both hepatic and intestinal forms. The species prevalence was expressed as a percentage and was determined by dividing the positively observed samples by the total number of positive samples. The species was identified morphologically then compared among species prevalence using analysis of variance at α=5%.

## Results and Discussion

The results of our study show that the prevalence of *Eimeria* spp. varied, i.e., Kulonprogo 68.6% (172/251), Bantul 78.38% (184/234), and Sleman 63.79% (171/265). The prevalence of rabbit coccidiosis in all investigated areas was 70.26% (527/750). In this study, we observed both hepatic and intestinal coccidiosis in Indonesian rabbits.

**Figure-1:**
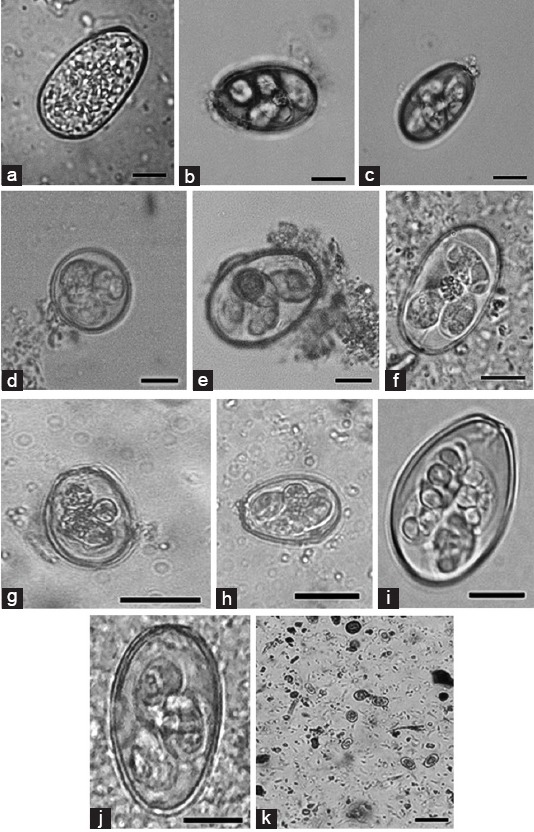
*Eimeria* spp. commonly circulate in Indonesia. (a) Unsporulated *Eimeria stiedae* from liver tissue squash, (b) *Eimeria flavescens*, (c) *Eimeria coecicola*, (d) *Eimeria exigua*, (e) *Eimeria media*, (f) *Eimeria perforans*, (g) *Eimeria intestinalis*, (h) *Eimeria magna*, (i) *Eimeria piriformis*, (j) *Eimeria irresidua*, (k) multiple species infection in a specimen. Scale bar=10 µm.

**Figure-2:**
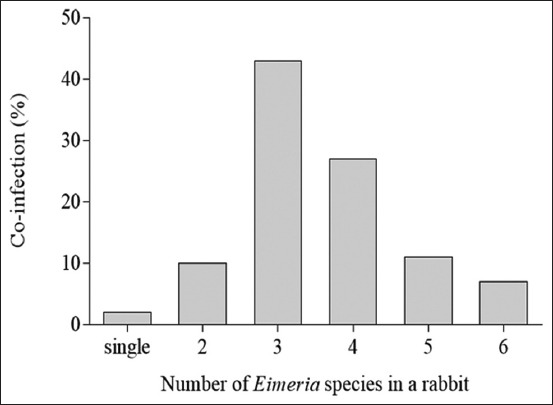
The percentage of co-infection between *Eimeria* spp. species which occurs in the rabbit population from Yogyakarta.

**Figure-3:**
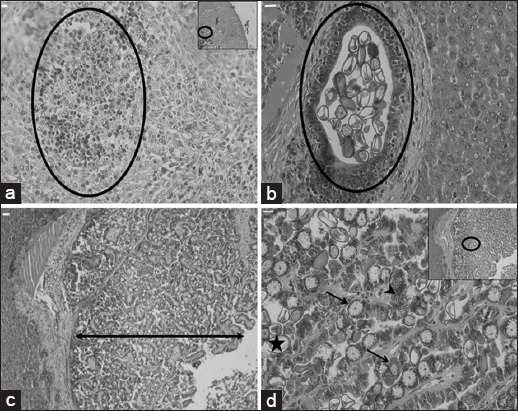
Severe hepatic coccidiosis lesion as indicated. (a) A complex of inflammatory cells in liver tissue (circle), (b) oocysts develop within the epithelium of the biliary duct and are detected in the biliary duct (circle), (c) massive proliferation of the epithelial line of the biliary duct (double-headed arrow), (d) intracellular development of *Eimeria stiedae*, macro- and micro-gamonts (arrows), schizogony process (arrowhead), developed oocyst (star). Scale bar=10 µm.

Of the 17 *Eimeria* species that infect rabbits [9], we found that 10 species were circulating among investigated rabbits. Ten species of *Eimeria* spp. were identified from the infected samples (n=527) in this study, i.e., *Eimeria flavescens* (80%, 421/527), *Eimeria coeciola* (78%, 411/527), *Eimeria perforans* (61%, 322/527), *Eimeria exigua* (37%, 195/527), *Eimeria media* (33%, 173/527), *E. stiedae* (31%, 165/527), *Eimeria irresidua* (12%, 63/527), *Eimeria magna* (11%, 58/527), *Eimeria intestinalis* (10%, 53/527), and *Eimeria piriformis* (10%, 53/527) (Figure-1). The prevalence was highest for *E. flavescens* and *E. coeciola* compared with the other *Eimeria* spp. (p≤0.0001). We also found specimens with co-infection of *Eimeria* spp. between single to 6 species in the positive samples. In total, specimens were co-infected with 1, 2, 3, 4, 5 and 6 species at 2, 10, 43, 27, 11 and 7% respectively (Figure-2). Rabbits with hepatic coccidiosis showed infiltration primarily of eosinophils and other polymorphonuclear cells in the liver parenchyma (Figure-3a) followed by severe inflammation around the biliary duct where *E. stiedae* developed (Figure-3b). Various developmental stages of *E. stiedae* were found within the biliary duct with a massive epithelial cell proliferation that was clearly observed (Figure-3b-d).

Recently, rabbits became popular as pet animals as well as meat sources in Indonesia. Although the rabbit industry is not as big as that for poultry or cattle, rabbit production is assumed to become more profitable in upcoming years [3]. Since the awareness of consuming non-red types of meat is increasing, rabbit meat is being considered as a delicacy and a healthy food product. In addition, it is easy to digest for daily consumption by both children and older adults [1]. However, information about rabbit diseases, especially coccidiosis, is still limited among farmers. To the best of our knowledge, we provide the first information about rabbit coccidiosis in several areas investigated on Java Island.

Rabbit coccidiosis is reported worldwide and affects different rabbit types with serious economic losses as consequences. Coccidiosis occurs both in meat and fur types of rabbits in China, which supply more than 40% of the world’s rabbit demands, with infection reaching 56.4% and *E. perforans* as the most prevalent [14]. *E. perforans* and *E. flavescens* were reportedly the two common species infecting wild rabbits in France [15]. In Egypt, the prevalence of natural infection among rabbit populations reached 70% with *E. intestinalis* and *E. coecicola* as predominant species [16]. Both intestinal and hepatic coccidiosis was also determined as the dominant parasitological cases (78.83%) during the veterinary hygiene inspection in Poland [17].

The fecal condition in this investigation was divided into two categories, namely, hard fecal dropping/grapes-like cecotropes (75%, 563/750) and watery-very soft cecotropes (25%, 187/750). The presence of watery cecotropes is higher in young rabbits <3 months old (67%, 125/187) compared with older groups more than 3 months old in common (33%, 62/187). However, *Eimeria* spp., in this paper, did not always detect in these abnormal fecal conditions. Of 38% (200/527) of infected rabbits were aged <3 months and another 62% (327/527) infection occurred in rabbits more than 3 months old. Rabbit at <3 months old group were categorized as suckling and small-sized rabbits since there were no precise record of rabbit age from the farmers. Of 88% rabbits infected by *Eimeria* spp. in this report (464/527) did not show any clinical signs of gastrointestinal disorder such as bloat distended stomach or lethargy. The McMaster count of 257 positive samples showed that OPG ≤1000 was found in 68%, ≤5000 was in 25%, and ≥10,000 was in 7%. Since individual species of rabbit coccidia differ in their pathogenicity [18] and we did not separate the amount of the pathogenic from the non-pathogenic *Eimeria* species in mixed infections, we could not analyze the correlation of the species composition and the presence of pathological signs if rabbits were infected.

Besides specific and curative treatments for rabbit coccidiosis, several prevention strategies can be used to minimize the number of coccidiosis cases. The rabbit’s biosecurity management by removing fecal oocysts sooner than they finish sporulation to be infective reduces the number of infective oocytes. However, the farmer cannot rely on the fecal disposal alone but must use anticoccidial drugs mixed in the food pellets or drinking water, the most common preventive method [19]. Although it still requires intense research and development, vaccination using a precocious line of agents seems to be quite promising for potential application in the near future [10,11,18].

## Conclusion

*Eimeria* spp. is detected in high prevalence among rabbit in Indonesia with commonly occurs in mixed infections. We describe *Eimeria* spp. that are circulating in Indonesia, provide information to both farmers and veterinarians, and suggest the significance of rabbit coccidiosis. To the best of our knowledge, we provide the first information about rabbit coccidiosis in Indonesia.

## Authors’ Contributions

PHH, YPK, and SP performed the experiments and analyzed the data. PHH and SP designed the study, coordinated the work, and wrote the manuscript. All authors read and approved the final manuscript.
